# Developing Organizational Requirements to Standardize Delivery and Improve Quality of Acute Leukemia Care in Ontario

**DOI:** 10.3390/curroncol31080347

**Published:** 2024-08-15

**Authors:** Leslie Verville, Cassandra McKay, Tom Kouroukis, Suzanna Apostolovski, Mitchell Sabloff, Rena Buckstein, Kardi Kennedy, Karen Yee, Amanda Eakins, Christopher Bredeson

**Affiliations:** 1 Cancer Programs, Ontario Health (Cancer Care Ontario), Toronto, ON M5G 2L3, Canada; cassandra.mckay@ontariohealth.ca (C.M.); kourouk@hhsc.ca (T.K.); suzanna.apostolovski@ontariohealth.ca (S.A.); amanda.eakins@ontariohealth.ca (A.E.); cbredeson@toh.ca (C.B.); 2 Hamilton Health Sciences, Hamilton, ON L8N 3Z5, Canada; 3 Division of Hematology, Department of Medicine, University of Ottawa, Ottawa, ON K1N 6N5, Canada; msabloff@toh.ca; 4 The Ottawa Hospital Research Institute, Ottawa, ON K1N 6N5, Canada; 5 Odette Cancer Centre, Sunnybrook Health Sciences Centre, Toronto, ON M4N 3M5, Canada; rena.buckstein@sunnybrook.ca; 6 Kingston Health Sciences Centre, Kingston, ON K7L 2V7, Canada; kardi.kennedy@kingstonhsc.ca; 7 Princess Margaret Cancer Centre, Toronto, ON M5G 2M9, Canada; karen.yee@uhn.ca

**Keywords:** acute myeloid leukemia, acute lymphoblastic leukemia, health policy, organization and administration

## Abstract

Acute leukemia is a rapidly progressive cancer of the blood and bone marrow that requires a high degree of complex, specialized, resource-intensive clinical and supportive care. The aging Canadian population has introduced an unprecedented demand on the health care system for a variety of illnesses, including acute leukemia. The purpose of this work was to develop organizational requirements for service providers delivering care for patients aged 18 years and older with acute leukemia within a single-payer health care system in Ontario. This initiative was intended to support streamlining high-quality health care across Ontario. We worked collaboratively with an expert panel to conduct a review of the literature to synthesize the organizational requirements for delivering acute leukemia care. A total of 229 requirements were developed. The requirements were categorized into themes including (1) facility requirements, including infrastructure, data management, safety, policies and procedures; (2) availability of clinical services and service complexity; (3) personnel, including roles, responsibilities, and ongoing education; (4) patient care; (5) quality management; (6) clinical research; and (7) laboratory services. These requirements will act as a framework for the provision of service, complexity of care, safety, accessibility, and quality care across all levels from the patient, organization, and system perspectives. This framework will help support person-centred care, emphasizing providing care close to home, while optimizing the use of specialized resources. Moving forward, Ontario Health (Cancer Care Ontario) will continue to work with acute leukemia service providers in the province to determine compliance and focus improvement efforts in priority areas.

## 1. Introduction

Acute leukemia is a rapidly progressive cancer of the blood and bone marrow; requiring a high degree of complex, specialized, resource-intensive care which may include chemotherapy, hematopoietic stem cell transplantation and varying degrees of supportive care.

The aging Canadian population has introduced an unprecedented demand on the health care system for a variety of illnesses, including acute leukemia. Appropriate planning for this unique patient population is crucial and necessary to meet this need and must consider several critical components, including a service delivery model that is multidisciplinary, shared across care settings, and is person-centred, all of which must be supported by a transparent funding model and include measurement and performance management strategies to drive system improvements.

The Canadian single-payer health care system is a model of universal health coverage that is publicly funded and administered by the federal, provincial, and territorial governments. The federal government provides funding to the provinces and territories who are then responsible for organizing, delivering, and regulating health care services within their jurisdictions. The Ontario health system provides comprehensive cancer care through a hub-and-spoke model of regional cancer centres, large general hospitals, and smaller community hospitals [1].

Ontario Health (Cancer Care Ontario) is an agency created by the Government of Ontario to connect, coordinate, and transform our province’s health care system. Ontario Health works with partners, providers, and patients to ensure that everyone in Ontario has equitable access to high-quality care, when and where they need it. This provincial agency oversees the coordination of cancer services across the province. This oversight spans the entire spectrum of cancer care including prevention, screening, diagnosis, treatment, survivorship, and palliative care. Ontario Health (Cancer Care Ontario) also supports the improvement of cancer services, ensuring that people have access to high-quality, timely, and equitable cancer care anywhere in Ontario. The Complex Malignant Hematology (CMH) Program, managed by Ontario Health (Cancer Care Ontario), oversees the delivery of acute leukemia care, stem cell transplant, and chimeric antigen receptor therapy. The CMH Program aims to ensure that patients have access to high-quality, safe, and appropriate services across the province and that these services are aligned with best practices and standards.

In 2017, Cancer Care Ontario released the Acute Leukemia Provincial Plan which provides an overview of how adult acute leukemia services should be defined, organized, and delivered across Ontario [2]. The plan outlined strategic goals and objectives to improve the delivery of coordinated, high-quality, and timely care as close to home as possible. The plan focuses on better patient outcomes and experiences by providing evidence-informed clinical care. The establishment of provincial oversight in this area of health care is aimed to support the system to reach these goals.

Performance criteria, such as organizational standards or requirements, can support expectations and quality assurance across public health service providers [3,4]. Organizational standards are valuable tools that help make informed decisions and enhance the quality and effectiveness of programs and services as well as helping to identify resource and human health resource (HHR) gaps [3]. These standards aid in prioritizing and allocating resources, identifying necessary changes in policies or program directions to meet objectives, and informing leadership about operational modifications for improved efficiency [3,4,5,6].

The purpose of this work was to develop organizational requirements for service providers delivering care for patients aged 18 years and older with acute leukemia in Ontario. The organizational requirements serve to provide a framework for planning and delivery of consistent, safe, and evidence-informed care at existing and new acute leukemia service provider sites across the province.

## 2. Setting

Ontario is a large and diverse province, home to approximately 14.6 million residents. The province encompasses 14 regional cancer programs, each with a host hospital that serves as a regional cancer centre responsible for delivering cancer services to the population within their catchment area, in collaboration with other hospitals, and community care providers. The regional cancer programs vary in size, scope, and capacity, depending on the needs and resources of their regions. Some programs provide specialized services, such as stem cell transplantation and gynecological oncology services, while others offer standard services such as systemic therapy or radiation treatment and refer patients to other centres for more complex treatments. At this time, there are ten centres across Ontario that provide the full spectrum of acute leukemia services.

**Target Population:** This work is targeted to the provision of acute leukemia care within the adult setting, defined as patients 18 years and older, in Ontario.

**Intended Users:** This work has been developed for clinicians, other health care professionals, and hospital and system administrators involved in the planning and delivery of adult acute leukemia care at existing Transplant and Acute Leukemia Service Sites, Acute Leukemia Service Sites, and Acute Leukemia Shared-Care Partner Centres, as well as by those interested in becoming service provider sites.

## 3. Methods

We convened a working group consisting of hematologists, nurse practitioners, and health care administrators, under the guidance of Ontario Health (Cancer Care Ontario)’s Acute Leukemia Advisory Committee to oversee this initiative. A membership list can be found in the Supplementary Materials, Section S1. The role of this group was to (1) develop acute leukemia specifications for the organizational structure and clinical practice at acute leukemia services sites within the Ontario context, (2) assist in the development of dissemination and implementation strategies, and (3) act as champions for the developed specifications.

We conducted an environmental scan and a review of the literature to synthesize the organizational requirements for delivering acute leukemia care.

This work was built upon an existing guideline developed by the Program in Evidence-Based Care (PEBC), Ontario Health (Cancer Care Ontario), which demonstrates a practical framework and standards to guide the delivery of systemic treatment in Ontario [7]. We also used the publicly available Foundation for the Accreditation of Cellular Therapy (FACT) standards to guide the development and organization of our work. FACT is a North American organization that establishes evidence-based organizational standards for high-quality medical and laboratory practice in cellular therapies [8].

We used the PEBC Handbook to guide the reporting of this work [9].

At the time of this work, the province of Ontario had three levels of acute leukemia service providers which were distinguished based on the scope of the services they provided (Table 1). The acute leukemia requirements developed from this work were organized according to these three service types.

### 3.1. Defining Criteria

We developed a search strategy in consultation with a team of reference librarians (Cancer Care Ontario Library Services). The search strategy was developed with search terms relevant to the organization and delivery of acute leukemia care including ‘acute leukemia’, ‘hematology’, ‘recommendation’, ‘standard’, and ‘guideline’. The search was limited to publications within the previous 10 years of the search date (2007–2017) and to the English language. The complete search strategy can be found in the Supplementary Materials, Section S2.

We searched databases including the Guidelines International Network, National Guidelines Clearinghouse, Standards and Guidelines Evidence Inventory for Cancer Guidelines, Clinical Practice Guidelines Infobase and Trip guideline. We also searched the grey literature which included websites for the National Health System, including the National Institute for Health and Care Excellence (NICE), Scottish Intercollegiate Guidelines Network and Health Improvement Scotland, FACT, American Society of Clinical Oncology, American Society of Hematology, European Society for Medical Oncology, Alberta Health Services, Cancer Care Nova Scotia, British Columbia Cancer Agency, Cancer Care Manitoba, the Hospital for Sick Children, Pediatric Oncology Group of Ontario, Western Australian Department of Health, National Comprehensive Cancer Network, Belgian Health Care Knowledge Centre, European LeukemiaNet, Health Quality Ontario, Agency for Healthcare Research and Quality, and Cancer Care Ontario.

We selected literature based on the following inclusion and exclusion criteria (Table 2).

### 3.2. Literature Selection

We used a three-phase process to determine the relevancy of the literature. In the first phase, paired reviewers (members of the Cancer Care Ontario team) independently screened titles to determine possible relevancy. In the second phase, abstracts of possibly relevant articles were reviewed, followed by a full-text review. Discussion-based consensus occurred between each phase of the review process. Any disagreements between reviewers regarding relevancy were also resolved through discussion.

### 3.3. Extraction of Standards and Requirements

The requirements from the relevant literature were extracted from the source into a Microsoft Office Excel table for the working group to review. The table included the source of the requirement, the requirement as worded from the source, and the level of acute leukemia service provider with which the requirement pertained (if this information was known).

### 3.4. Synthesis and Analysis

The working group members were tasked with assessing each requirement to determine if it should be endorsed as is, adapted to meet the needs of adult patients with acute leukemia in Ontario, or deemed irrelevant/inappropriate for the current context. 

The working group also determined where there were gaps in the literature and where developing new requirements would be most appropriate. New requirements were proposed at working group meetings. Each newly proposed requirement was discussed and refined and, finally, agreed upon by the working group members.

We adopted the following nomenclature from FACT to describe the necessity of each requirement (Table 3) [10].

All requirements were further reviewed through a second and third round of reviews which involved members of the Acute Leukemia Advisory Committee, relevant Ontario Health (Cancer Care Ontario) Program administrators, Provincial Program Heads, senior leadership, as well as external clinical and administrative expert reviewers (Supplementary Materials, Section S1). As part of these rounds of review, requirements that were identified as being a responsibility of the hospital, rather than specific to the hospital’s Acute Leukemia Program, were removed; for example, requirements under the provision of Accreditation Canada whereby hospitals are generally required to follow to maintain accreditation in good standing. 

Informed by various sources including FACT, NICE, provincial standards, and committee member suggestions, requirements were categorized into groups relevant to the context of Ontario. The categories included (1) facility requirements, including infrastructure, data management, safety, and policies and procedures; (2) availability of clinical services and service complexity; (3) personnel, including roles, responsibilities, and ongoing education; (4) patient care; (5) quality management; (6) clinical research; and (7) laboratory services.

## 4. Results

**Figure 1:**
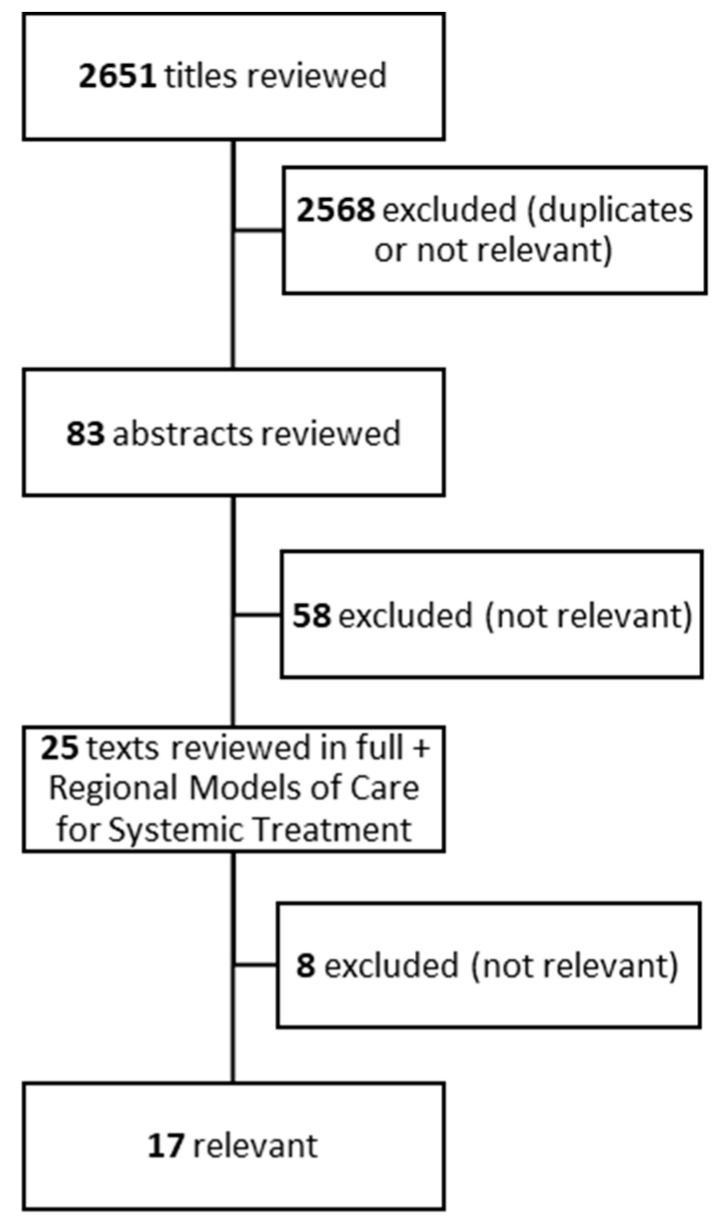
Flowchart of literature search.

Our literature search identified 2651 records. Of these, 2568 records were identified as either duplicates or deemed not relevant, leaving 83 records for abstract screening. In phase two, we screened 83 abstracts and 25 full-text articles in phase three. Finally, 17 reports were included (Figure 1).

**Figure 2:**
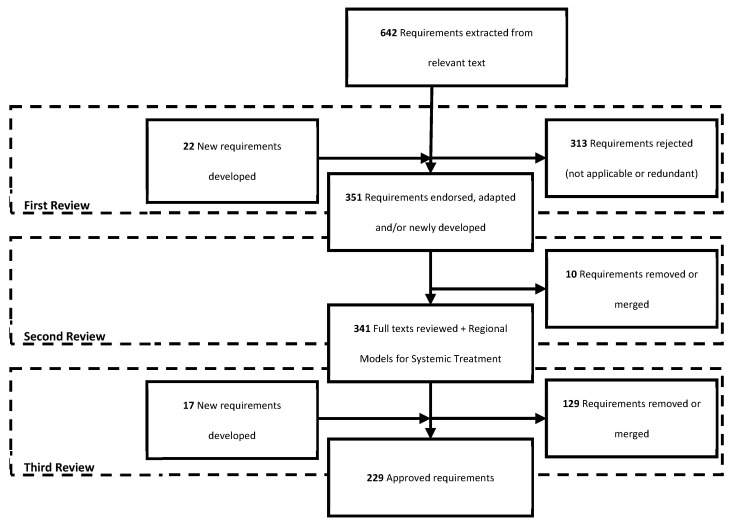
Flowchart of requirement review.

We extracted 642 requirements from the included reports. All requirements were reviewed and refined through a three-part process. During the first round of review by the working group, 313 requirements were rejected as they were determined not relevant to the local context or redundant of other requirements. In addition, 22 requirements were newly developed, determined to fill missing gaps in the literature. The second round of review removed an additional 10 requirements either rejected or merged with other requirements due to similarity. Finally, the third round of review relinquished an additional 129 requirements. At this time, 17 new requirements were generated, yielding a total of 229 requirements included in the final report (Table 4, Figure 2) [11,12,13,14,15,16,17,18,19,20,21,22,23,24,25,26,27,28,29,30,31,32,33].

Due to the lack of available guidelines and evidence, the requirements are largely based on expert opinion informed by the endorsement and adaptation of existing standards and requirements from other jurisdictions, including from Ontario Health (Cancer Care Ontario), NICE [34], and the Foundation for the Accreditation of Cellular Therapy (FACT) at the University of Nebraska Medical Centre [8]. New requirements were developed where the committee members determined gaps in the literature. The final provincial policy requirements report was completed and made publicly available on the Ontario Health (Cancer Care Ontario) website (cancercareontario.ca/en/guidelines-advice/types-of-cancer/69431).

**Table 4:** Organizational Requirements. (Organizational requirements checklist by site type is available in the Supplementary Materials, Section S3).

GENERAL	
**1.1**	The Centre **shall** have a clearly defined organizational structure [11].
**1.2**	The Clinical Program **shall** consist of an integrated medical team housed in a defined location(s), including a Clinical Program Medical Director(s), who is responsible for the medical aspects of the operation of the service, in collaboration with appropriate facility administrators. This includes the design of the diagnostic pathway, resource use, and reporting standards [11].
**1.2.1**	The Centre **should** consider the organization of current services to allow the development of disease-specific clinics where patient numbers are sufficient [12].
**1.3**	The Centre **shall** work to implement the current version of Complex Malignant Hematology Models of Care Recommendations [13].
**1.4**	The Clinical Program **shall** be located in a facility that is licensed, registered, or accredited by Accreditation Canada [14].
**1.5**	The Centre **shall** comply with the current versions of the Ontario Health (Cancer Care Ontario) standards for the delivery of systemic treatment including, but not limited to, the Regional Models of Care for Systemic Treatment: Standards for the Organization and Delivery of Systemic Treatment, as appropriate to their designated level of service [7].
**1.6**	The Centre **shall** comply with the current version of the Ontario Health (Cancer Care Ontario) guidelines for the safe administration of systemic therapy including, but not limited to, the following reports: Safe Administration of Chemotherapy: Safety During Chemotherapy Ordering, Transcribing, Dispensing, and Patient Identification [14,15]. Safe Administration of Systemic Cancer Therapy Part 2: Administration of Systemic Treatment and Management of Preventable Adverse Events [14,16].
**1.7**	The Centre **shall** participate as part of a Provincial Acute Leukemia Network developed by the Regional Cancer Programs in partnership with Ontario Health (Cancer Care Ontario) [2,14].
**1.7.1**	The Centres **shall** have clear and reliable systems (e.g., processes, tools) for communicating with relevant health care professionals at other service sites [11].
**1.7.2**	The Transplant and Acute Leukemia Service Sites and Acute Leukemia Service Sites **shall** provide mentorship (e.g., onsite training, sharing resources, availability to respond to questions, etc.) to affiliated Acute Leukemia Shared-Care Partner Centres, Systemic Treatment Hospitals, and other centres, as appropriate [2]. ^1^
**1.8**	The Clinical Program **shall** have a designated acute leukemia team that includes a Clinical Program Medical Director, a Quality Manager, and a total of at least three (3) full-time attending hematologists ^2^ providing 24 h coverage (including by phone) [2,11,17]. ^1^
**1.8.1**	Shared-care centres **shall** have access to acute leukemia expertise through an Acute Leukemia Service Site [2]. ^3^
**1.9**	The Centre **shall** collaboratively participate in provincial capacity management activities (e.g., CritiCall Ontario), as needed, to ensure access to timely care [2,14].
**1.10**	The Centre **should** provide clinical services for patients with hematological cancers delivered by multidisciplinary hemato-oncology teams [11].
**1.11**	The Centre **should** provide intensive induction therapy (including induction therapy following remission and subsequent relapse) or less intensive therapy with the intent of remission to a minimum of 10 patients with acute leukemia per year and who are at risk of more than 7 days of neutropenia (absolute neutrophil count of 0.5 × 10^9^/litre or lower) [11,14]. ^1^
**CLINICAL UNIT—INPATIENT**	
**2.1**	The Centre **shall** have an ICU or readily available access to an ICU [2,11].
**2.2**	The Centre **shall** provide patients who have acute leukemia and are at risk of more than 7 days of neutropenia (absolute neutrophil count of 0.5 × 10^9^/litre or lower) with an inpatient room with an occupancy of no greater than two (2) patients. The room should be equipped with its own bathroom [11,14].
**2.2.1**	If patients require isolation in accordance with local infectious disease practices, the patient **shall** be isolated in a private room with a private bathroom [14].
**2.3**	The Centre **shall** have a designated inpatient unit that minimizes airborne microbial contamination, in keeping with the Guideline for the Implementation of Air Standards in Ontario [18].
**2.4**	The Centre **shall** ensure that there are beds available in a dedicated ward within the hospital with the capacity to treat the planned volumes of patients, including the availability of a flex bed to allow for the direct, urgent admission of patients being managed on an outpatient basis [11,13].
**2.5**	The Centre **should** have the level of staffing required for febrile neutropenia patients that is equivalent to that in a high-acuity unit, as per hospital policies [11].
**CLINICAL UNIT—OUTPATIENT**	
**2.6**	The Centre **shall** provide monitoring following leukemia therapy in an ambulatory setting and ensure that there is an area for outpatient care that provides the following: Reasonably protects the patient from transmission of infectious agents and minimizes risk of airborne microbial contamination Allows for confidential examination and evaluation Provides, as necessary, an area for patient isolation, administration of intravenous infusions, multiple medications, and/or blood component transfusions [11,17].
**2.7**	The Centre **should** consider ambulatory care for patients who have hematological malignancies that are in remission and other clinically appropriate patients, who are at risk of more than 7 days of neutropenia (absolute neutrophil count of 0.5 × 10^9^/litre or lower) (i.e., outpatient consolidation chemotherapy, other less intensive therapies) [11,13].
**2.8**	The Clinical Program **should** account for the following when assessing patients to determine if ambulatory care is appropriate: **2.8.1** Access to appropriate and timely transport **2.8.2** Accommodation and communication facilities **2.8.3** Availability of caregiver to provide support **2.8.4** Comorbidities **2.8.5** Distance and travel times to treatment in case of neutropenic fever and other toxicities **2.8.6** Patient’s and/or caregivers’ understanding of the safety requirements of ambulatory care and their individual treatment plan **2.8.7** Patient preference [11].
**CLINICAL UNIT—SUPPORTIVE SERVICES**	
**2.9**	The Centre **shall** have an on-site blood bank with the ability to deliver packed red blood cells and platelet transfusions, as well as plasma and factor concentrates, without delay [2,11].
**2.9.1**	The Centre’s blood bank **should** have a record of patient transfusions that is accessible to the members of the multidisciplinary Clinical Program [14].
**2.10**	The Centre **shall** have 24 h access to irradiated blood products needed for the care of acute leukemia patients as per the National Advisory Committee on Blood and Blood Products—Recommendations for use of Irradiated Blood Components in Canada, 2018, or as updated [19].
**2.11**	The Clinical Program **shall** have dedicated pharmacists with oncology/hematology training involved in the inpatient and outpatient care of leukemia patients [2,11].
**2.12**	The Centre **shall** have 24 h availability of medications needed for the care of acute leukemia patients [17].
**2.13**	The Centre **shall** have appropriate diagnostic services to care for the acute leukemia patient population and complications of therapy, including, but not limited to, bronchoscopy, cross-sectional imaging, endoscopy, and renal support [11,14].
**2.14**	The Centre **shall** have access to expertise and supporting technologies for image-guided biopsy and interventional radiology/oncology [14].
**2.15**	The Centre **shall** have access to leukapheresis therapy [20].
**2.16**	The Centre **should** have expertise in vascular access for central venous catheter insertions [11].
**PERSONNEL**	
**3.1**	The Clinical Program **shall** include members of a multidisciplinary care team (which may include Clinical Associates, Nurse Practitioners, Physician Assistants, Registered Nurses, and other providers) with the appropriate training and oversight by a hematologist/oncologist [13].
**3.1.1**	The scope of responsibility of the multidisciplinary care team members **shall** be defined [17].
**3.1.2**	The Clinical Program Team (Physicians/Pharmacists/Nurse Practitioners/Physicians’ Assistants/Clinical Associates) **shall** participate in a minimum of ten (10) hours of educational activities (e.g., self-directed education, rounds, webinars, meetings, conferences) annually, related to acute leukemia care or management [17].
**CLINICAL PROGRAM MEDICAL DIRECTORS**	
**3.2**	The Clinical Program Medical Director **shall** have at least two (2) years of experience as an attending physician responsible for the direct clinical management of acute leukemia patients in the inpatient and outpatient settings [17]. ^1^
**3.3**	The Clinical Program Medical Director **shall** have oversight of the medical care provided by all members of the Clinical Program [17].
**3.4**	The Clinical Program Medical Director or designate **shall** be responsible for verifying the knowledge and skills of members of the Clinical Program multidisciplinary care team, including nurses, pharmacists, physicians, and other providers once every three (3) years [17].
**3.5**	Working in partnership with hospital administration, the Clinical Program Medical Director **shall** be responsible for administrative and clinical operations, including compliance with these recommendations and applicable laws and regulations [17].
**3.6**	Working in partnership with hospital administration, the Clinical Program Medical Director **shall** be responsible for all elements of the design of the Clinical Program including quality management as per Section D.4, whether internal or contracted services, which may be part of a broader malignant hematology program [17].
**CLINICAL PROGRAM MEDICAL DIRECTORS AND ATTENDING PHYSICIANS**	
**3.7**	Clinical Program Medical Directors and Attending Physicians **shall** have received specific training in each of the following areas as applicable to the Clinical Program’s services: **3.7.1** Applicable regulations and reporting responsibilities for adverse events and reactions, as required by Health Canada [17]. **3.7.2** Documentation and reporting for patients on investigational protocols and completion of Good Clinical Practice training as recognized by their institution [35].
**ATTENDING PHYSICIANS**	
**3.8**	Attending Physicians **shall** have received specific training in each of the following areas as applicable to the Clinical Program’s services: **3.8.1** Administration of acute leukemia therapy **3.8.2** Blood transfusion management **3.8.3** Cardiac dysfunction **3.8.4** Diagnosis and management of fungal disease **3.8.5** Diagnosis and management of infectious and non-infectious complications of acute leukemia therapy, including, but not limited to: **3.8.5.1** Appropriate antimicrobial prophylaxis **3.8.5.2** Hemophagocytosis **3.8.5.3** Hypersensitivity reactions **3.8.5.4** Management of neutropenia and neutropenic fever **3.8.5.5** Management of mucositis, nausea, and vomiting **3.8.5.6** Management of thrombocytopenia and bleeding, including recognition of disseminated intravascular coagulation **3.8.5.7** Monitoring and management of pain **3.8.5.8** Neurologic toxicity **3.8.5.9** Renal dysfunction **3.8.5.10** Respiratory distress **3.8.5.11** Tumour lysis and cytokine release syndrome **3.8.6** Evaluation of post-leukemia therapy outcomes and late effects **3.8.7** Goals of care **3.8.8** Indications and appropriateness of leukemia therapy, including appropriate selection of suitable candidates for HCT or cellular therapy referral **3.8.9** Palliative and end-of-life care **3.8.10** Survivorship care **3.8.11** Use of irradiated blood products, where appropriate [14,17].
**3.9**	Attending physicians **shall** each have had a minimum total of one (1) year of supervised training in the management of acute leukemia patients in both inpatient and outpatient settings [17].
**PHYSICIANS’ ASSISTANTS, CLINICAL ASSOCIATES, NURSE PRACTITIONERS, AND REGISTERED NURSES**	
**3.10**	Physicians’ Assistants, Clinical Associates, Nurse Practitioners, and Registered Nurses **shall** have received specific training and maintain competence in the acute leukemia-related skills that they routinely practice within their respective role including: **3.10.1** Administration of acute leukemia therapy **3.10.2** Administration of blood products, growth factors, and other supportive therapies **3.10.3** Care interventions to manage acute leukemia therapy-related complications, including, but not limited to: **3.10.3.1** Cardiac dysfunction **3.10.3.2** Cytokine release syndrome **3.10.3.3** Disseminated intravascular coagulation **3.10.3.4** Hypersensitivity reactions **3.10.3.5** Infectious processes **3.10.3.6** Mucositis **3.10.3.7** Nausea and vomiting **3.10.3.8** Neurologic toxicity **3.10.3.9** Neutropenic fever **3.10.3.10** Pain management **3.10.3.11** Renal and hepatic failure **3.10.3.12** Respiratory distress **3.10.3.13** Tumor lysis syndrome **3.10.4** Palliative and end-of-life care **3.10.5** Survivorship care [2,14,17].
**3.11**	The Clinical Program **shall** have a sufficient number of nurses, based on number of patients and acuity, appropriately trained in the care of acute leukemia patients [17,21].
**3.12**	The Clinical Program **should** have specialized oncology nurses with national certification in oncology through the Canadian Nurses Association and additional knowledge, clinical skills, and clinical decision-making in leukemia (such as training from the de Souza institute) [7].
**PHARMACISTS**	
**3.13**	Training and knowledge of designated pharmacists **shall** include: **3.13.1** Requirements detailed in the Regional Models of Care for Systemic Treatment: Standards for the Organization and Delivery of Systemic Treatment, as appropriate to their designated level of service [7,14]. **3.13.2** Hematology/oncology patient care, including the role of, administration of, and complications of systemic therapy for acute leukemia patients [17]. **3.13.3** Monitoring for and recognition of drug/drug and drug/food interactions and necessary dose modifications [17]. **3.13.4** Recognition of medications that require adjustment for organ dysfunction [17]. **3.13.5** Therapeutic drug monitoring, including, but not limited to, anti-infective agents, immunosuppressive agents, anti-seizure medications, and anticoagulants [17].
**3.14**	Clinical pharmacists, or designate, **shall** work with the multidisciplinary team to perform medication reconciliation, monitor for side effects, including medication side effects, and provide supportive care and manage symptoms [2].
**OTHER SPECIALISTS**	
**3.15**	The Clinical Program **shall** have access to certified or trained consulting specialists and/or specialist groups from key disciplines capable of assisting in the management of acute leukemia patients, including, but not limited to: **3.15.1** Cardiology **3.15.2** Dentistry **3.15.3** Dermatology **3.15.4** Gastroenterology **3.15.5** Infectious Disease **3.15.6** Intensive Care **3.15.7** Nephrology **3.15.8** Neurology **3.15.9** Obstetrics/Gynecology **3.15.10** Ophthalmology **3.15.11** Pain and Symptom Management **3.15.12** Palliative and End-of-Life Care **3.15.13** Pathology and Hematopathology (including molecular diagnostics and genetics) **3.15.14** Physiatry/Rehabilitation Medicine **3.15.15** Psychiatry **3.15.16** Pulmonary Medicine **3.15.17** Radiology, including relevant subspecialty expertise related to: **3.15.17.1** Cross-sectional Imaging **3.15.17.2** Interventional Radiology **3.15.18** Radiation Oncology **3.15.19** Surgical services that includes general surgery, thoracic, neurosurgery, and ears, nose, and throat (ENT) surgery **3.15.20** Transfusion Medicine [2,14,17].
**3.16**	The Clinical Program **shall** have access to a multidisciplinary care team, including designated staff with appropriate training and education, to assist in the provision of pre-treatment evaluation, treatment, and post-treatment follow-up and care. Designated staff/roles **shall** include: **3.16.1** Data management staff sufficient to comply with Sections titled ‘Data Management’ and ‘Laboratory Services’ **3.16.2** Decision-support resources to collate and analyze quality indicators **3.16.3** Dietitian **3.16.4** Interpretative/translation services **3.16.5** Patient care coordinator **3.16.6** Physical therapy and occupational therapy **3.16.7** Psychology **3.16.8** Social work **3.16.9** Speech language pathology **3.16.10** Spiritual care ^4^ [2,14,17].
**QUALITY MANAGERS**	
**3.17**	There **shall** be a Clinical Program Quality Manager to establish and maintain systems to review, modify, and approve all policies and SOPs intended to monitor compliance with these recommendations or the performance of the Clinical Program [17].
**3.18**	The Clinical Program Quality Manager **shall** participate in a minimum of ten (10) hours of educational activities (e.g., self-directed education, rounds, webinars, meetings, conferences), annually, related to acute leukemia therapy and/or quality management [17].
**QUALITY MANAGEMENT**	
**4.1**	Centres **shall** have a Quality Management Program that allows the Clinical Program Medical Director and all members of the care team to maintain their competency as internally assessed by the Clinical Program Medical Director or designate. The clinical competency of the Clinical Program Medical Director should be assessed by another identified staff member [17].
**4.1.1**	The Clinical Program Medical Director or designate **shall** have authority over and responsibility for ensuring that the overall Quality Management Program is effectively established and maintained [11,17].
**4.1.2**	The Clinical Program Medical Director or designate **shall** review the Quality Management activities with representatives in key positions in all elements of the Clinical Program, at a minimum, quarterly [17].
**4.1.2.1**	Key performance data and review findings **shall** be reported to staff [17].
**4.1.2.2**	Meetings **should** have defined attendees, documented minutes, and assigned actions [17].
**4.1.2.3**	In the course of their regular meetings, the Clinical Program **should** annually review patient feedback of the acute leukemia program and any actions implemented, and improvement programs [22].
**4.1.3**	The Clinical Program Medical Director or designate **shall** annually review the effectiveness of the overall Quality Management Program [17].
**4.1.4**	The Clinical Program Medical Director or designate **shall not** have oversight of his/her own work if this person also performs other tasks in the Clinical Program [17].
**4.2**	The Clinical Program **shall** establish and maintain a written Quality Management Plan [17].
**4.2.1**	The Clinical Program Medical Director or designate **shall** be responsible for the Quality Management Plan [17].
**4.2.2**	The Quality Management Plan **shall** include, or summarize and reference, a comprehensive system for document control [17].
**4.2.2.1**	There **shall** be policies or SOPs for the development, approval, implementation, distribution, review, revision, and archival of all critical documents [17].
**4.2.3**	The Quality Management Plan **shall** include, or summarize and reference, policies and SOPs for the establishment and maintenance of written agreements [17].
**4.2.3.1**	Agreements **shall** be established with external parties (who are accredited, as appropriate) providing critical services that could affect the quality and safety of care for patients in the Clinical Program [17].
**4.2.3.2**	Agreements **shall** be dated and reviewed on a regular basis [14].
**4.2.4**	The Quality Management Plan **shall** include, or summarize and reference, policies and SOPs for occurrences including near misses, errors, accidents, deviations, adverse events, adverse reactions, and complaints. This may be the same as existing policy at the centre [17].
**4.2.5**	The Quality Management Plan **shall** include, or summarize and reference, policies and SOPs for actions to take in the event that the Clinical Program’s operations are interrupted [17].
**4.2.6**	The Quality Management Plan **shall** include, or summarize and reference, policies and SOPs for qualification of critical manufacturers, vendors, equipment, supplies, reagents, facilities, and services [17].
**4.2.6.1**	Qualification plans, results, and reports **shall** be reviewed and approved by the Quality Manager and Clinical Program Medical Director or designate [17].
**4.2.7**	The Quality Management Plan **shall** include, or summarize and reference, policies and SOPs for the evaluation of risk in changes to a process to confirm that the changes do not create an adverse impact or inherent risk elsewhere in the operation [17].
**4.2.8**	The Quality Management Plan **shall** include, or summarize and reference, an organizational chart of key positions and functions within the Clinical Program (governance structure) [17].
**4.2.8.1**	The Quality Management Plan **shall** include a description of how these key positions interact to implement the quality management activities [17].
**4.2.8.2**	The Quality Management Plan **shall** include, or summarize and reference, policies and SOPs addressing personnel requirements for each key position in the Clinical Program. Personnel requirements shall include at a minimum:
**4.2.8.2.1**	A current job description for all staff [17].
**4.2.8.2.2**	A system to document the following for all staff: Initial qualifications New employee orientation Initial training, competency, and retraining when appropriate for all procedures performed Continued competency for each critical function performed, assessed annually at a minimum continuing education [17].
**4.2.9**	The Quality Management Plan **shall** include key performance indicators and outcome analysis [14].
**4.2.9.1**	The Clinical Program **should** work with Ontario Health (Cancer Care Ontario) to meet Provincial Acute Leukemia Program benchmarks, including: **4.2.9.1.1** Consult Wait Times **4.2.9.1.2** Length of stay **4.2.9.1.3** Mortality **4.2.9.1.4** Survival Outcomes **4.2.9.1.5** Treatment Utilization **4.2.9.1.6** Treatment Wait Times, and **4.2.9.1.7** Other indicators, as established [14].
**4.2.9.2**	In addition to the Ontario Health (Cancer Care Ontario) recommended metrics, review of outcome analysis **shall** include at a minimum: **4.2.9.2.1** Central venous catheter infection and/or thrombosis **4.2.9.2.2** Complete remission **4.2.9.2.3** Hospital acquired infections **4.2.9.2.4** ICU admissions [14,17].
**4.2.10**	The Quality Management Plan **shall** include, or summarize and reference, policies and SOPs for, and a schedule of, audits of the Clinical Program’s activities to verify compliance with elements of the Quality Management Program and policies and SOPs, applicable laws or regulations, and these Specifications [17].
**4.2.10.1**	The results of audits **shall** be used to recognize problems, detect trends, identify improvement opportunities, and implement corrective and preventive actions, when necessary, and follow up on the effectiveness of these actions in a timely manner [17].
**4.2.10.2**	Audits **shall** be conducted by an individual with sufficient expertise to identify problems, but who is not solely responsible for the process being audited [17].
**POLICIES AND PROCEDURES**	
**5.1**	The Centre **shall** have SOPs that are detailed, as per hospital’s policy, to allow qualified staff to follow and complete the procedures successfully [14].
**5.2**	The Clinical Program **shall** have SOPs defining local protocols for patient eligibility and selection for care (including performance status, prognostic factors, comorbidities) and consent [11,17].
**5.3**	There **shall** be written SOPs or guidelines, including, but not limited to: **5.3.1** All clinical procedures **5.3.2** Administration of systemic therapy **5.3.3** Central venous access device care **5.3.4** Management of complications with systemic therapy: **5.3.4.1** Nausea, vomiting, pain, and other discomforts **5.3.4.2** Monitoring of blood counts and transfusion of blood products **5.3.4.3** Monitoring and management of infections and use of antimicrobials **5.3.4.4** Monitoring of organ dysfunction or failure **5.3.5** Prophylaxis, management and care of immunocompromised patients [11,14,17,23].
**5.4**	The Centre **shall** have policies addressing safe administration of patient-specific radiation therapy [24].
**5.5**	The Clinical Program **shall** have policies or SOPs in place for planned discharges and provision of follow-up care post-systemic therapy care, including transfer of patient, if required [17].
**5.6**	The Clinical Program **shall** have a SOP for inter-institutional patient transfer that specifies clinical criteria for eligibility to transfer the patient and information transferred with the patient [14].
**5.7**	The Clinical Program **shall** have an SOP for electronic decision-making tools used by the Clinical Program documenting the tools development, validation, and auditing [14].
**5.8**	Staff training and, if appropriate, competency **shall** be documented before performing a new or revised SOP or guideline [17].
**5.9**	Planned deviations from SOPs **shall** be pre-approved by the Clinical Program Medical Director, or designate, and reviewed by the Quality Manager [17].
**5.10**	The Centre **should** have a SOP for the recognition of systemic therapy-related complications and emergencies requiring rapid notification of the Clinical Program [17].
**5.11**	The Centre **should** have an institutional SOP for direct admission of patients to the hematology ward or other facilities equipped to rapidly assess and manage potentially life-threatening complications of systemic therapy (such as neutropenic sepsis or bleeding), where appropriate [11].
**5.12**	The Clinical Program **should** have an established framework or policies for the transfer of patients to the ICU, as appropriate. The framework/policies should include written guidelines for communication, patient monitoring, and prompt triage or transfer of patients to an ICU when appropriate [17,25].
**5.13**	The Centre **should** have written policies for communication with the person’s primary care physician and other teams involved in treatment [11].
**PATIENT CARE**	
**6.1**	The Clinical Program **shall** obtain patient informed consent, as per Accreditation Canada, for systemic therapy, which is documented in the patient’s medical record by a licensed health care professional familiar with the proposed systemic therapy [7,14].
**6.1.1**	The Clinical Program **shall** provide information regarding the risks, benefits, and alternatives of the proposed systemic therapy [17].
**6.1.2**	The Centre **shall** provide the patient with information regarding the impact of treatment on fertility and information, including contact information, about fertility preservation [14].
**6.2**	The Centre **shall** provide the patient with access to active palliative care, supportive end-of-life care [26], and medical assistance in dying (MAID) [27]. In accordance with patient and family’s wishes, this care could be provided at centres at and beyond the acute leukemia service provider sites and can be offered closer to home [2].
**6.3**	If radiation is used, the centre **shall** document a final report with details of the radiation therapy administered in the patient’s medical record that is accessible to the acute leukemia team [17].
**6.4**	The Clinical Program **shall** provide services for adolescents and young adult patients and a process describing the transition and acceptance of adolescents and young adult patients to adult care, as appropriate [12,17].
**6.5**	The Centre **should** provide formal Multidisciplinary Case Conferences (MCC), where acute leukemia cases may be presented and discussed, attended by individuals detailed in the Cancer Care Ontario’s MCC Standards [11,28].
**6.6**	The Clinical Program **should** provide acute leukemia patients with access to a designated contact person, as part of a multidisciplinary team, throughout the duration of their care [29].
**6.7**	The Centre **should** provide care in alignment with Cancer Care Ontario’s Person-Centred Care Guidelines [30], in efforts to meet the Person-Centred goals and objectives detailed in the most recent version of the Ontario Cancer Plan [31].
**CLINICAL RESEARCH**	
**7.1**	The Centre **shall** conduct and/or provide access to clinical trials and consider available clinical trials when assessing patient treatment options [2,22]. ^1^
**7.2**	The Centre **shall** report available clinical trials for patients with acute leukemia to Ontario Health (Cancer Care Ontario) (by emailing OH-CCO_SSOInfo@ontariohealth.ca) or a central repository to inform other centres of their availability [14]. ^1^
**DATA MANAGEMENT**	
**8.1**	The Centre **shall** be compliant with laws and regulations regarding the storage and use of personal health information as detailed by the Information and Privacy Commissioner of Ontario [14,15].
**8.2**	The Centre **shall** be compliant with the Cancer Care Ontario Data Book [2].
**8.3**	The Clinical Program **shall** collect all the data necessary to complete data submission requirements of Ontario Health (Cancer Care Ontario), as detailed in the Funding Agreement with Ontario Health (Cancer Care Ontario) [14].
**8.4**	The Clinical Program **should** have an IT system that allows: **8.4.1** Specimen booking and registration at source **8.4.2** Input and update of clinical information **8.4.3** Integrated/synoptic reporting **8.4.4** Secure internal and external two-way communication between health care professionals [11].
**8.5**	Defined data management staff should participate in continuing education annually [17].
**LABORATORY SERVICES**	
**9.1**	The Centre **shall** ensure that patients have access to all required pathology and molecular diagnostic tests as listed in the most recent version of the Consensus Pathology Recommendation for Complex Malignant Hematology Report [24].
**9.1.1**	Testing sites **shall** meet all relevant Institute for Quality Management in Healthcare requirements and maintain Institute for Quality Management certification [14].
**9.1.2**	All testing performed for clinical management **should** be licensed and performed by accredited labs [14].
**9.1.3**	Testing sites performing cytogenetics and molecular diagnostics **shall** meet provincial turnaround time targets [32].
**9.2**	The Centre **shall** classify and report acute leukemia and subtypes based on the current World Health Organization classification system and Ontario Health (Cancer Care Ontario) Synoptic Reporting, when in place [11,32,33].

^1^ Does not apply to Shared-care Partner Centres. ^2^ May include the Clinical Program Medical Director if the Clinical Program Medical Director is a hematologist. ^3^ Does not apply to Transplant and Acute Leukemia Service Sites. ^4^ Although Spiritual Care is not included as part of the Acute Leukemia Funding Model, access should be provided as requested by patients.

## 5. Discussion

A strategic priority of the Ontario Provincial Cancer Plan is to improve access to high-quality cancer care for all Ontarians, regardless of where they live throughout the province. This means providing care closer to home whenever possible, ensuring that patients, of various characteristic backgrounds, needing specialized or complex care can receive the appropriate level of expertise and resources. For patients with acute leukemia, this can be challenging as the diagnosis and treatment of this disease often requires specialized diagnostic and prognostic laboratory testing, drugs, and procedures that are very costly and urgently needed, provided by highly trained, specialized health care professionals, and are not widely available across the province. 

Inspired by FACT, which sets standards for the quality and safety of cellular therapy and accredits centres that meet those standards, we wanted to apply a similar approach to acute leukemia care in Ontario, as this is a complex and rapidly evolving field that requires specialized skills and resources. Therefore, Ontario Health (Cancer Care Ontario), in collaboration with regional cancer programs and other stakeholders, have developed a series of organizational requirements for acute leukemia service providers delivering care in Ontario. We conducted a review of the literature to synthesize current organizational standards and developed additional standards when gaps were identified; in the end, we defined 229 organizational requirements. 

The intent of this work is not to penalize centres, but rather to support service providers as they work toward meeting the requirements, striving to improve the delivery of high-quality care to patients with acute leukemia. The organizational requirements, as a framework, aim to help cancer centres assess their current infrastructure and readiness to provide care, identify gaps and opportunities for improvement, and develop a road map to achieve their desired level of care. The framework supports consistency of care across sites while also supporting an integrated provincial network of health care across all levels and regions.

A significant benefit of the requirements report is supporting cancer centres to pursue the necessary resources and support to implement plans and ensure quality and safety of care. By using the criteria and indicators in the report, sites can demonstrate their current level of performance, their alignment with provincial requirements and guidelines, and their potential to deliver higher-level care if supported by additional resources. This way, the report can serve as a valuable tool for advocacy and resource mobilization for sites that aspire to improve their acute leukemia care.

We recognize that developing organizational requirements is only the first step in the process of quality improvement and that the successful implementation and evaluation of these requirements depends on many factors, such as leadership, engagement, communication, education, funding, and infrastructure. We highly recommend that organizational requirements are implemented in collaboration with relevant stakeholders to facilitate their adoption and integration into daily practice and to monitor and measure their impact on the quality and safety of care.

### 5.1. Strengths and Limitations 

Strengths: The requirements are derived from a comprehensive literature review and environmental scan of the current state of acute leukemia care in Ontario and other jurisdictions, which provides a foundational evidence base for the requirements. We used an integrated knowledge translation approach throughout the development of our work as it was guided by a provincial advisory committee and a working group consisting of experts and stakeholders from various disciplines and regions, ensuring the relevance and applicability of the requirements to the Ontario context. Using this approach, we ensured end-user buy-in of the final report. Our work offers practical and feasible requirements for sites to improve their infrastructure and quality of care for acute leukemia patients. Finally, the requirements can be used as a framework for assessing and monitoring the performance of sites that offer acute leukemia care, using criteria and indicators that reflect best practices and provincial standards and guidelines. 

Limitations: The requirements are drawn from a variety of sources including the expert opinion of clinical and administrative stakeholders from across the province. These requirements have not been validated in an acute leukemia setting. In addition, their applicability and generalizability to different populations is unknown. In addition, we acknowledge that some of the requirements may not be feasible or applicable in all settings. Secondly, the requirements were based upon available data and information at the time of its development, which may not always reflect the most recent evidence or practice. Therefore, the report should be updated and revised periodically to incorporate new knowledge, emerging evidence, and changes in clinical practice. Another limitation of our work is that we did not exhaustively explore all clinical aspects of care. While these are equally important and interrelated components of quality care, they are beyond the scope of this project. We hope that our work will complement and inform the development of clinical practice guidelines and pathways for acute leukemia patients and that together they will provide a comprehensive and coherent framework for improving the outcomes and experiences of this patient group.

### 5.2. Next Steps and Future Directions 

**Figure 3:**
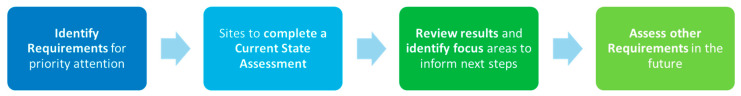
Implementation of organizational requirements.

Relevant organizational requirements should be implemented via a staged approach. Our suggested approach is outlined in Figure 3. In collaboration with stakeholders, a subset of requirements of highest priority should be identified. Priority requirements should be used as a baseline for sites interested in becoming service sites. Sites should complete self-assessments to determine which requirements are currently being met and which they should work toward. Finally, using a collaborative approach, sites should be supported in their ambitions for growth in achieving additional organizational requirements. 

Ontario Health (Cancer Care Ontario) will continue to work with cancer centres across Ontario, in addition to our advisory committee and other applicable stakeholders, to determine and monitor the application of the requirements. Together, we aim to identify areas of focus, inform next steps and support improvement through implementation of these criteria. An example of this future work includes adapting organizational requirements for emerging therapies such as the developing area of highly effective low-intensity therapies which will be administered by many more centres than the current ten.

As the literature surrounding acute leukemia care is ever evolving, it is highly recommended that the organizational requirements presented in this report be reviewed for completeness and accuracy, at a minimum, every three years. Future iterations of this work should consider other guidance released by Ontario Health (Cancer Care Ontario), including guidance related to health equity and the provision of culturally appropriate care.

## 6. Conclusions

In this paper, we have presented a set of requirements for improving the quality and safety of acute leukemia care in Ontario, based on the best available evidence and expert consensus. These requirements cover various aspects of care delivery, such as diagnosis, treatment, supportive care, and follow-up. We have also outlined the next steps for assessing and monitoring the implementation of these requirements, which involve working collaboratively with service providers and stakeholders to identify gaps, challenges, and opportunities for improvement. Our work aims to support the provision of high-quality care for patients with acute leukemia across Ontario and to foster a culture of continuous learning and improvement in this field. We hope that our work will also be useful for other jurisdictions that are interested in enhancing their acute leukemia services and outcomes.

## Figures and Tables

**Table 1:** Acute Leukemia Service Sites and Services.

Service Site	Services Provided
Transplant and Acute Leukemia Service Site	Provides the full scope of acute leukemia services and stem cell transplant. These sites may also provide chimeric antigen receptor T-cell (CAR T-cell) therapy. The Transplant and Acute Leukemia Service Site acts as the hub of activity for patients with complex hematologic malignancies. Across the province, these centres work together to ensure a standardized approach across the care continuum.
Acute Leukemia Service Site	Provides the full scope of acute leukemia services, including intensive induction therapy and less intensive therapy, with the intent of achieving remission and disease control, as well as post-remission treatment and care. The Acute Leukemia Service Sites do not perform stem cell transplant or CAR T-cell therapy but instead work with the Transplant Sites to ensure that their patients have access to these services. They may also work with Transplant Sites to accept autologous stem cell transplant patients for post-transplant recovery closer to home. The Acute Leukemia Service Site may partner with an Acute Leukemia Shared Care Partner Centre and/or other systemic treatment hospitals to support care closer to home.
Acute Leukemia Shared-Care Partner Centre	Provides a subset of services for patients through a shared-care model. Partner centres work with an Acute Leukemia Service Site, or a Transplant and Acute Leukemia Service Site, to share portions of care on an ongoing basis and/or accept autologous transplant patients for post-transplant recovery closer to home.

**Table 2:** Inclusion and exclusion criteria.

Population	Adults (Defined as ≥ 18 Years of Age) Canada, the United States, the United Kingdom, Australia, Europe
Disease Type	Acute leukemia, Acute Myeloid Leukemia (AML), Acute erythroblastic leukemia, acute myelomonocytic leukemia, hematology Exclusion: angiomyolipoma, cord blood, venous thromboembolism, lupus
Study Designs	Guideline, recommendations, standards, organizational standards, practice guidelines, practice parameters, consensus standards, systematic review
Study Characteristics and Timeline	Exclusion: clinical trial, randomized clinical trial English language, published within the previous 10 years of the search date (2007–2017)

**Table 3:** Recommended Nomenclature.

Shall	to be complied with at all times
Should	an activity is recommended or advised, but for which there may be appropriate alternatives
May	permissive and is used primarily for clarity

## Data Availability

The datasets generated during and/or analyzed during the current study are available from the corresponding author on reasonable request.

## Supplementary Materials

The following supporting information can be downloaded at: https://www.mdpi.com/article/10.3390/curroncol31080347/s1, Section S1: Members of the Acute Leukemia Specifications Working Group; Section S2: Search Strategy. Section S3: Organizational Requirements Checklist.
