# Supplementary material for: Developing Organizational Requirements to Standardize Delivery and Improve Quality of Acute Leukemia Care in Ontario
Source: Curr Oncol. 2024 Aug 15;31(8):4656–74. doi: 10.3390/curroncol31080347 (PMC11352428; doi:10.3390/curroncol31080347)
Supplement: Supplementary file 1 [file curroncol-31-00347-s001.zip › curroncol-3150606-supplementary.pdf]

Supplemental File

Section S1

Members of the Acute Leukemia Specifications Working Group

Disclaimer: Membership roles and associations are reported as they were at the time the working group was convened in 2018. Roles and associations may not be reflective of current affiliations.

Name	Role(s)	Potential Conflicts of Interest
Dr. Christopher Bredeson	Chair, Clinical Lead, Quality Care and Access, Complex Malignant Hematology, Cancer Care Ontario Hematologist, The Ottawa Hospital	No relevant conflicts of interest to declare.
Dr. Rena Buckstein	Hematologist, Sunnybrook Health Sciences Centre	Research funding from Celgene.
Kardi Kennedy	Program Operational Director & Regional Director, Cancer Program, Kingston Health Sciences Centre	No relevant conflicts of interest to declare.
Dr. Tom Kouroukis	Provincial Head, Complex Malignant Hematology, Cancer Care Ontario Hematologist, Hamilton Health Sciences Corporation	No relevant conflicts of interest to declare.
Lia Kutzscher	Nurse Practitioner, Royal Victoria Regional Health Centre	No relevant conflicts of interest to declare.
Dr. Janet MacEachern	Hematologist, Grand River Hospital	No relevant conflicts of interest to declare.
Kit McCann	Lead Nurse Practitioner, Windsor Regional Hospital	No relevant conflicts of interest to declare.
Dr. Mitchell Sabloff	Hematologist, Director of Leukemia Program, The Ottawa Hospital	Research support from Sanofi Canada (2016/17). Honoraria from Pfizer Canada (Oct 2018), Celgene (Jun 2018), Jazz Pharmaceuticals (Apr 2018), and Novartis (Feb 2017/18).
Dr. Karen Yee	Hematologist, Princess Margaret Cancer Centre – University Health Network	Ad board and research funding from various pharmaceutical companies.
Sherrie Hertz	Group Manager, Specialized Services Oversight Program, Cancer Care Ontario	No relevant conflicts of interest to declare.
Cassandra McKay	Lead, Specialized Services Oversight Program, Cancer Care Ontario	No relevant conflicts of interest to declare.
Amanda Wong	Lead (Acting), Specialized Services Oversight Program, Cancer Care Ontario	No relevant conflicts of interest to declare.
Suzanna Apostolovski	Coordinator, Specialized Services Oversight Program, Cancer Care Ontario	No relevant conflicts of interest to declare.

*Cancer Care Ontario is now a part of Ontario Health (Cancer Care Ontario)

Section S2

Search Strategy

Primary Literature Search (performed by Cancer Care Ontario Library Services):
Subject: Development of organizational requirements for acute leukemia
Date range: 10 years
Language(s): English
Database(s): Medline, Embase
Medline Search Strategy:
Set 1 Acute Leukemia/Hematology
exp Leukemia, Myeloid, Acute/ or exp Leukemia, Erythroblastic, Acute/ or exp Leukemia, Myelomonocytic, Acute/
or
(Acute adj leuk?emia$ adj2 (granulocytic or megakaryocytic or monocytic or myeloblastic or myelomonocytic or
myeloid or myelocytic or myelogenous or nonlymphoblastic or non-lymphoblastic or nonlymphocytic or nonlymphocytic or erythroid or monoblastic or basophilic or erythroid or monoblastic or nonlymphoid or nonlymphoid)).ti,ab,kw.
or
(AML not (angiomyol: or amylose or amlodipine)).ti,ab
or
*Hematology/ or (hematology or haematology or haematologic or hematologic).ti,ab
Not
(Cord blood or Venous Thromboembolism or lupus* or prostat*).ti,ab,kw
And
Set 2 Guidelines/Recommendations/Standards
*Practice Guidelines as Topic/ or guideline.pt.
or
(organizational standard$ or organisational standard$ or practice parameter$ or practice guideline$).tw
or (guideline or guidelines or recommended or recommendation or recommend or consensus or standards).ti. or
(guideline or guidelines or recommended or recommendation or recommend or consensus or standards).kw
Medline search history as run
Ovid MEDLINE(R) Epub Ahead of Print, In-Process & Other Non-Indexed Citations, Ovid MEDLINE(R) Daily and Ovid
MEDLINE(R) <1946 to Present>

Section S3

Organizational Requirements Checklist

#	Recommendation	Transplant and Acute Leukemia Service Site	Acute Leukemia Service Site	Shared-Care Partner Centre
**GENERAL**				
**1.1**	The Centre **shall** have a clearly defined organizational structure.(11)	Yes	Yes	Yes
**1.2**	The Clinical Program **shall** consist of an integrated medical team housed in a defined location(s), including a Clinical Program Medical Director(s), who is responsible for the medical aspects of the operation of the service, in collaboration with appropriate facility administrators. This includes the design of the diagnostic pathway, resource use and reporting standards.(11)	Yes	Yes	Yes
**1.2.1**	The Centre **should** consider the organization of current services to allow the development of disease specific clinics where patient numbers are sufficient.(12)	Yes	Yes	Yes
**1.3**	The Centre **shall** work to implement the current version of Complex Malignant Hematology Models of Care Recommendations.(13)	Yes	Yes	Yes
**1.4**	The Clinical Program **shall** be located in a facility that is licensed, registered, or accredited by Accreditation Canada.(14)	Yes	Yes	Yes
**1.5**	The Centre **shall** comply with the current versions of the Ontario Health (Cancer Care Ontario) standards for the delivery of systemic treatment, including but not limited to, the Regional Models of Care for Systemic Treatment: Standards for the Organization and Delivery of Systemic Treatment, as appropriate to their designated level of service.(7)	Yes	Yes	Yes
**1.6**	The Centre **shall** comply with the current version of the Ontario Health (Cancer Care Ontario) guidelines for the safe administration of systemic therapy, including but not limited to, the following reports:<br>• Safe Administration of Chemotherapy: Safety During Chemotherapy Ordering, Transcribing, Dispensing, and Patient Identification.(14,15)<br><br>Safe Administration of Systemic Cancer Therapy Part 2: Administration of Systemic Treatment and Management of Preventable Adverse Events.(14,16)	Yes	Yes	Yes

#	Recommendation	Transplant and Acute Leukemia Service Site	Acute Leukemia Service Site	Shared-Care Partner Centre
**1.7**	The Centre **shall** participate as part of a Provincial Acute Leukemia Network developed by the Regional Cancer Programs in partnership with Ontario Health (Cancer Care Ontario).(2,14)	Yes	Yes	Yes
**1.7.1**	The Centres **shall** have clear and reliable systems (e.g., processes, tools) for communicating with relevant health care professionals at other service sites.(11)	Yes	Yes	Yes
**1.7.2**	The Transplant and Acute Leukemia Service Sites and Acute Leukemia Service Sites **shall** provide mentorship (e.g., onsite training, sharing resources, availability to respond to questions, etc.) to affiliated Acute Leukemia Shared-Care Partner Centres, Systemic Treatment Hospitals and other centres, as appropriate.(2)	Yes	Yes	N/A
**1.8**	The Clinical Program **shall** have a designated acute leukemia team that includes a Clinical Program Medical Director, a Quality Manager, and a total of at least three (3) full time attending hematologists[1], providing 24-hour coverage (including by phone).(2,11,17)	Yes	Yes	No
**1.8.1**	Shared-care centres **shall** have access to acute leukemia expertise through an Acute Leukemia Service Site.(2)	N/A	N/A	Yes
**1.9**	The Centre **shall** collaboratively participate in provincial capacity management activities (e.g., CritiCall Ontario), as needed to ensure access to timely care.(2,14)	Yes	Yes	Yes
**1.10**	The Centre **should** provide clinical services for patients with hematological cancers delivered by multidisciplinary hemato-oncology teams.(11)	Yes	Yes	Yes
**1.11**	The Centre **should** provide intensive induction therapy (including induction therapy following remission and subsequent relapse) or less intensive therapy with the intent of remission to a minimum of 10 patients with acute leukemia per year and who are at risk of more than 7 days of neutropenia (absolute neutrophil count of 0.5×10^9/litre or lower).(11,14)	Yes	Yes	No
**CLINICAL UNIT – INPATIENT**				
**2.1**	The Centre **shall** have an ICU or readily available access to an ICU.(2,17)	Yes	Yes	Yes
**2.2**	The Centre **shall** provide patients who have acute leukemia and are at risk of more than 7 days of neutropenia (absolute neutrophil count of 0.5×10^9/litre or lower) with an inpatient	Yes	Yes	Yes

[1] May include the Clinical Program Medical Director if the Clinical Program Medical Director is a hematologist.

#	Recommendation	Transplant and Acute Leukemia Service Site	Acute Leukemia Service Site	Shared-Care Partner Centre
	room with an occupancy of no greater than two (2) patients. The room **should** be equipped with its own bathroom.(11,14)			
**2.2.1**	If patients require isolation in accordance with local infectious disease practices, the patient **shall** be isolated in a private room with a private bathroom.(14)	Yes	Yes	Yes
**2.3**	The Centre **shall** have a designated inpatient unit that minimizes airborne microbial contamination, in keeping with the Guideline for the Implementation of Air Standards in Ontario.(18)	Yes	Yes	Yes
**2.4**	The Centre **shall** ensure there are beds available in a dedicated ward within the hospital with the capacity to treat the planned volumes of patients, including the availability of a flex bed to allow for the direct, urgent admission of patients being managed on an outpatient basis.(11,13)	Yes	Yes	Yes
**2.5**	The Centre **should** have the level of staffing required for febrile neutropenia patients that is equivalent to that in a high acuity unit, as per hospital policies.(11)	Yes	Yes	Yes
**CLINICAL UNIT – OUTPATIENT**				
**2.6**	The Centre **shall** provide monitoring following leukemia therapy in an ambulatory setting and ensure that there is an area for outpatient care that provides the following:<br>• Reasonably protects the patient from transmission of infectious agents and minimizes risk of airborne microbial contamination<br>• Allows for confidential examination and evaluation<br>• Provides, as necessary, an area for patient isolation, administration of intravenous infusions, multiple medications, and/or blood component transfusions.(11,17)	Yes	Yes	Yes
**2.7**	The Centre **should** consider ambulatory care for patients who have hematological malignancies that are in remission and other clinically appropriate patients, who are at risk of more than 7 days of neutropenia (absolute neutrophil count of 0.5×10^9/litre or lower) (i.e., outpatient consolidation chemotherapy, other less intensive therapies).(11,13)	Yes	Yes	Yes
**2.8**	The Clinical Program **should** account for the following when assessing patients to determine if ambulatory care is appropriate:	Yes	Yes	Yes
**2.8.1**	Access to appropriate and timely transport	Yes	Yes	Yes
**2.8.2**	Accommodation and communication facilities	Yes	Yes	Yes

#	Recommendation	Transplant and Acute Leukemia Service Site	Acute Leukemia Service Site	Shared-Care Partner Centre
**2.8.3**	Availability of caregiver to provide support	Yes	Yes	Yes
**2.8.4**	Comorbidities	Yes	Yes	Yes
**2.8.5**	Distance and travel times to treatment in case of neutropenic fever and other toxicities	Yes	Yes	Yes
**2.8.6**	Patient's and/or caregivers' understanding of the safety requirements of ambulatory care and their individual treatment plan	Yes	Yes	Yes
**2.8.7**	Patient preference.(11)	Yes	Yes	Yes
**CLINICAL UNIT - SUPPORTIVE SERVICES**				
**2.9**	The Centre **shall** have an on-site blood bank with ability to deliver packed red blood cells and platelet transfusions, as well as plasma and factor concentrates, without delay.(2,11)	Yes	Yes	Yes
**2.9.1**	The Centre's blood bank **should** have a record of patient transfusions that is accessible to the members of the multidisciplinary Clinical Program.(14)	Yes	Yes	Yes
**2.10**	The Centre **shall** have 24-hour access to irradiated blood products needed for the care of acute leukemia patients as per National Advisory Committee on Blood and Blood Products - Recommendations for use of Irradiated Blood Components in Canada, 2018, or as updated.(19)	Yes	Yes	Yes
**2.11**	The Clinical Program **shall** have dedicated pharmacists with oncology/hematology training involved in the inpatient and outpatient care of leukemia patients.(2,17)	Yes	Yes	Yes
**2.12**	The Centre **shall** have 24-hour availability of medications needed for the care of acute leukemia patients.(17)	Yes	Yes	Yes
**2.13**	The Centre **shall** have appropriate diagnostic services to care for the acute leukemia patient population and complications of therapy, including, but not limited to, bronchoscopy, cross-sectional imaging, endoscopy, and renal support.(11,14)	Yes	Yes	Yes
**2.14**	The Centre **shall** have access to expertise and supporting technologies for image-guided biopsy and interventional radiology/oncology.(14)	Yes	Yes	Yes
**2.15**	The Centre **shall** have access to leukapheresis therapy.(20)	Yes	Yes	Yes
**2.16**	The Centre **should** have expertise in vascular access for central venous catheter insertions.(11)	Yes	Yes	Yes
**PERSONNEL**				

#	Recommendation	Transplant and Acute Leukemia Service Site	Acute Leukemia Service Site	Shared-Care Partner Centre
**3.1**	The Clinical Program **shall** include members of multidisciplinary care team (which may include Clinical Associates, Nurse Practitioners, Physician Assistants, Registered Nurses and other providers) with the appropriate training and oversight by a hematologist/oncologist.(13)	Yes	Yes	Yes
**3.1.1**	The scope of responsibility of the multidisciplinary care team members **shall** be defined.(17)	Yes	Yes	Yes
**3.1.2**	The Clinical Program Team (Physicians/ Pharmacists/ Nurse Practitioners/ Physicians Assistants/ Clinical Associates) **shall** participate in a minimum of ten (10) hours of educational activities (e.g., self-directed education, rounds, webinars, meetings, conferences), annually, related to acute leukemia care or management.(17)	Yes	Yes	Yes[2]
**CLINICAL PROGRAM MEDICAL DIRECTORS**				
**3.2**	The Clinical Program Medical Director **shall** have at least two (2) years of experience as an attending physician responsible for the direct clinical management of acute leukemia patients in the inpatient and outpatient settings.(17)	Yes	Yes	No
**3.3**	The Clinical Program Medical Director **shall** have oversight of the medical care provided by all members of the Clinical Program.(17)	Yes	Yes	Yes
**3.4**	The Clinical Program Medical Director or designate **shall** be responsible for verifying the knowledge and skills of members of the Clinical Program multidisciplinary care team, including nurses, pharmacists physicians, and other providers once every three (3) years.(17)	Yes	Yes	Yes
**3.5**	Working in partnership with hospital administration, the Clinical Program Medical Director **shall** be responsible for administrative and clinical operations, including compliance with these recommendations and applicable laws and regulations.(17)	Yes	Yes	Yes
**3.6**	Working in partnership with hospital administration, the Clinical Program Medical Director **shall** be responsible for all elements of the design of the Clinical Program including quality management as per Section D.4, whether internal or contracted services, which may be part of broader malignant hematology program.(17)	Yes	Yes	Yes
**CLINICAL PROGRAM MEDICAL DIRECTORS AND ATTENDING PHYSICIANS**				

[2] At Shared-Care Partner Centres, Physicians/ Pharmacists/ Nurse Practitioners/ Physicians Assistants/ Clinical Associates should (rather than shall) participate in a minimum of ten (10) hours of educational activities (e.g., self-directed education, rounds, webinars, meetings, conferences), annually, related to acute leukemia care or management.

#	Recommendation	Transplant and Acute Leukemia Service Site	Acute Leukemia Service Site	Shared-Care Partner Centre
**3.7**	Clinical Program Medical Directors and Attending Physicians **shall** have received specific training in each of the following areas as applicable to the Clinical Program's services:	Yes	Yes	Yes
**3.7.1**	Applicable regulations and reporting responsibilities for adverse events and reactions, as required by Health Canada.(17)	Yes	Yes	Yes
**3.7.2**	Documentation and reporting for patients on investigational protocols and completion of Good Clinical Practice training as recognized by their institution.(21)	Yes	Yes	Yes
**ATTENDING PHYSICIANS**				
**3.8**	Attending Physicians **shall** have received specific training in each of the following areas as applicable to the Clinical Program's services:	Yes	Yes	Yes
**3.8.1**	Administration of acute leukemia therapy	Yes	Yes	Yes
**3.8.2**	Blood transfusion management	Yes	Yes	Yes
**3.8.3**	Cardiac dysfunction	Yes	Yes	Yes
**3.8.4**	Diagnosis and management of fungal disease	Yes	Yes	Yes
**3.8.5**	Diagnosis and management of infectious and non-infectious complications of acute leukemia therapy, including but not limited to:	Yes	Yes	Yes
**3.8.5.1**	Appropriate antimicrobial prophylaxis	Yes	Yes	Yes
**3.8.5.2**	Hemophagocytosis	Yes	Yes	Yes
**3.8.5.3**	Hypersensitivity reactions	Yes	Yes	Yes
**3.8.5.4**	Management of neutropenia and neutropenic fever	Yes	Yes	Yes
**3.8.5.5**	Management of mucositis, nausea, and vomiting	Yes	Yes	Yes
**3.8.5.6**	Management of thrombocytopenia and bleeding, including recognition of disseminated intravascular coagulation	Yes	Yes	Yes
**3.8.5.7**	Monitoring and management of pain	Yes	Yes	Yes
**3.8.5.8**	Neurologic toxicity	Yes	Yes	Yes
**3.8.5.9**	Renal dysfunction	Yes	Yes	Yes
**3.8.5.10**	Respiratory distress	Yes	Yes	Yes
**3.8.5.11**	Tumour lysis and cytokine release syndrome	Yes	Yes	Yes
**3.8.6**	Evaluation of post-leukemia therapy outcomes and late effects	Yes	Yes	Yes
**3.8.7**	Goals of care	Yes	Yes	Yes

#	Recommendation	Transplant and Acute Leukemia Service Site	Acute Leukemia Service Site	Shared-Care Partner Centre
**3.8.8**	Indications and appropriateness of leukemia therapy, including appropriate selection of suitable candidates for HCT or cellular therapy referral	Yes	Yes	Yes
**3.8.9**	Palliative and end of life care	Yes	Yes	Yes
**3.8.10**	Survivorship care	Yes	Yes	Yes
**3.8.11**	Use of irradiated blood products, where appropriate.(14,17)	Yes	Yes	Yes
**3.9**	Attending physicians **shall** each have had a minimum total of one (1) year of supervised training in the management of acute leukemia patients in both inpatient and outpatient settings.(17)	Yes	Yes	No
**PHYSICIANS ASSISTANTS, CLINICAL ASSOCIATES, NURSE PRACTITIONERS AND REGISTERED NURSES**				
**3.10**	Physicians Assistants, Clinical Associates, Nurse Practitioners and Registered Nurses **shall** have received specific training and maintain competence in the acute leukemia-related skills that they routinely practice within their respective role including:	Yes	Yes	Yes
**3.10.1**	Administration of acute leukemia therapy.	Yes	Yes	Yes
**3.10.2**	Administration of blood products, growth factors, and other supportive therapies.	Yes	Yes	Yes
**3.10.3**	Care interventions to manage acute leukemia therapy-related complications, including, but not limited to:	Yes	Yes	Yes
**3.10.3.1**	Cardiac dysfunction	Yes	Yes	Yes
**3.10.3.2**	Cytokine release syndrome	Yes	Yes	Yes
**3.10.3.3**	Disseminated intravascular coagulation	Yes	Yes	Yes
**3.10.3.4**	Hypersensitivity reactions	Yes	Yes	Yes
**3.10.3.5**	Infectious processes	Yes	Yes	Yes
**3.10.3.6**	Mucositis	Yes	Yes	Yes
**3.10.3.7**	Nausea and vomiting	Yes	Yes	Yes
**3.10.3.8**	Neurologic toxicity	Yes	Yes	Yes
**3.10.3.9**	Neutropenic fever	Yes	Yes	Yes
**3.10.3.10**	Pain management	Yes	Yes	Yes
**3.10.3.11**	Renal and hepatic failure	Yes	Yes	Yes
**3.10.3.12**	Respiratory distress	Yes	Yes	Yes
**3.10.3.13**	Tumor lysis syndrome	Yes	Yes	Yes

#	Recommendation	Transplant and Acute Leukemia Service Site	Acute Leukemia Service Site	Shared-Care Partner Centre
**3.10.4**	Palliative and end of life care	Yes	Yes	Yes
**3.10.5**	Survivorship care.(2,14,17)	Yes	Yes	Yes
**3.11**	The Clinical Program **shall** have a sufficient number of nurses, based on number of patients and acuity, appropriately trained in the care of acute leukemia patients.(17,22)	Yes	Yes	Yes
**3.12**	The Clinical Program **should** have specialized oncology nurses with national certification in oncology through the Canadian Nurses Association and additional knowledge, clinical skills and clinical decision making in leukemia (such as training from the de Souza institute).(7)	Yes	Yes	Yes
**PHARMACISTS**				
**3.13**	Training and knowledge of designated pharmacists **shall** include:	Yes	Yes	Yes
**3.13.1**	Requirements detailed in the Regional Models of Care for Systemic Treatment: Standards for the Organization and Delivery of Systemic Treatment, as appropriate to their designated level of service.(7,14)	Yes	Yes	Yes
**3.13.2**	Hematology/oncology patient care, including the role of, administration of and complications of systemic therapy for acute leukemia patients.(17)	Yes	Yes	Yes
**3.13.3**	Monitoring for and recognition of drug/drug and drug/food interactions and necessary dose modifications.(17)	Yes	Yes	Yes
**3.13.4**	Recognition of medications that require adjustment for organ dysfunction.(17)	Yes	Yes	Yes
**3.13.5**	Therapeutic drug monitoring, including, but not limited to, anti-infective agents, immunosuppressive agents, anti-seizure medications, and anticoagulants.(17)	Yes	Yes	Yes
**3.14**	Clinical pharmacists, or designate, **shall** work with the multidisciplinary team to perform medication reconciliation, monitor for side effects, including medication side effects, and provide supportive care and manage symptoms.(2)	Yes	Yes	Yes
**OTHER SPECIALISTS**				
**3.15**	The Clinical Program **shall** have access to certified or trained consulting specialists and/or specialist groups from key disciplines capable of assisting in the management of acute leukemia patients, including, but not limited to:	Yes	Yes	Yes
**3.15.1**	Cardiology	Yes	Yes	Yes
**3.15.2**	Dentistry	Yes	Yes	Yes
**3.15.3**	Dermatology	Yes	Yes	Yes

#	Recommendation	Transplant and Acute Leukemia Service Site	Acute Leukemia Service Site	Shared-Care Partner Centre
**3.15.4**	Gastroenterology	Yes	Yes	Yes
**3.15.5**	Infectious Disease	Yes	Yes	Yes
**3.15.6**	Intensive Care	Yes	Yes	Yes
**3.15.7**	Nephrology	Yes	Yes	Yes
**3.15.8**	Neurology	Yes	Yes	Yes
**3.15.9**	Obstetrics/Gynecology	Yes	Yes	Yes
**3.15.10**	Ophthalmology	Yes	Yes	Yes
**3.15.11**	Pain and Symptom Management	Yes	Yes	Yes
**3.15.12**	Palliative and End of Life Care	Yes	Yes	Yes
**3.15.13**	Pathology and Hematopathology (including molecular diagnostics and genetics)	Yes	Yes	Yes
**3.15.14**	Physiatry/Rehabilitation Medicine	Yes	Yes	Yes
**3.15.15**	Psychiatry	Yes	Yes	Yes
**3.15.16**	Pulmonary Medicine	Yes	Yes	Yes
**3.15.17**	Radiology, including relevant subspecialty expertise related to:	Yes	Yes	Yes
**3.15.17.1**	Cross-sectional Imaging	Yes	Yes	Yes
**3.15.17.2**	Interventional Radiology	Yes	Yes	Yes
**3.15.18**	Radiation Oncology	Yes	Yes	Yes
**3.15.19**	Surgical services that includes general surgery, thoracic, neurosurgery, and ears, nose, and throat (ENT) surgery	Yes	Yes	Yes
**3.15.20**	Transfusion Medicine.(2,14,17)	Yes	Yes	Yes
**3.16**	The Clinical Program **shall** have access to a multidisciplinary care team, including designated staff with appropriate training and education to assist in the provision of pre-treatment evaluation, treatment, and post-treatment follow-up and care. Designated staff/roles **shall** include:	Yes	Yes	Yes
**3.16.1**	Data management staff sufficient to comply with Section D.8 and D.9	Yes	Yes	Yes
**3.16.2**	Decision-support resources to collate and analyze quality indicators	Yes	Yes	Yes
**3.16.3**	Dietitian	Yes	Yes	Yes
**3.16.4**	Interpretative/translation services	Yes	Yes	Yes
**3.16.5**	Patient care coordinator	Yes	Yes	Yes

#	Recommendation	Transplant and Acute Leukemia Service Site	Acute Leukemia Service Site	Shared-Care Partner Centre
**3.16.6**	Physical therapy and occupational therapy	Yes	Yes	Yes
**3.16.7**	Psychology	Yes	Yes	Yes
**3.16.8**	Social work	Yes	Yes	Yes
**3.16.9**	Speech language pathology	Yes	Yes	Yes
**3.16.10**	Spiritual care[3].(2,14,17)	Yes	Yes	Yes
**QUALITY MANAGERS**				
**3.17**	There **shall** be a Clinical Program Quality Manager to establish and maintain systems to review, modify, and approve all policies and SOPs intended to monitor compliance with these recommendations or the performance of the Clinical Program.(17)	Yes	Yes	Yes
**3.18**	The Clinical Program Quality Manager **shall** participate in a minimum of ten (10) hours of educational activities (e.g., self-directed education, rounds, webinars, meetings, conferences), annually, related to acute leukemia therapy and/or quality management.(17)	Yes	Yes	Yes
**QUALITY MANAGEMENT**				
**4.1**	Centres **shall** have a Quality Management Program that allows Clinical Program Medical Director and all members of the care team to maintain their competency as internally assessed by the Clinical Program Medical Director or designate. The clinical competency of the Clinical Program Medical Director **should** be assessed by another identified staff member.(17)	Yes	Yes	Yes
**4.1.1**	The Clinical Program Medical Director or designate **shall** have authority over and responsibility for ensuring that the overall Quality Management Program is effectively established and maintained.(11,17)	Yes	Yes	Yes
**4.1.2**	The Clinical Program Medical Director or designate **shall** review the Quality Management activities with representatives in key positions in all elements of the Clinical Program, at a minimum, quarterly.(17)	Yes	Yes	Yes
**4.1.2.1**	Key performance data and review findings **shall** be reported to staff.(17)	Yes	Yes	Yes
**4.1.2.2**	Meetings **should** have defined attendees, documented minutes, and assigned actions.(17)	Yes	Yes	Yes

[3] Although Spiritual Care is not included as part of the Acute Leukemia Funding Model, access should be provided as requested by patients.

#	Recommendation	Transplant and Acute Leukemia Service Site	Acute Leukemia Service Site	Shared-Care Partner Centre
**4.1.2.3**	In the course of their regular meetings, the Clinical Program **should** annually review patient feedback of the acute leukemia program and any actions implemented, and improvement programs.(23)	Yes	Yes	Yes
**4.1.3**	The Clinical Program Medical Director or designate **shall** annually review the effectiveness of the overall Quality Management Program.(17)	Yes	Yes	Yes
**4.1.4**	The Clinical Program Medical Director or designate **shall not** have oversight of his/her own work if this person also performs other tasks in the Clinical Program.(17)	Yes	Yes	Yes
**4.2**	The Clinical Program **shall** establish and maintain a written Quality Management Plan.(17)	Yes	Yes	Yes
**4.2.1**	The Clinical Program Medical Director or designate **shall** be responsible for the Quality Management Plan.(17)	Yes	Yes	Yes
**4.2.2**	The Quality Management Plan **shall** include, or summarize and reference, a comprehensive system for document control.(17)	Yes	Yes	Yes
**4.2.2.1**	There **shall** be policies or SOPs for the development, approval, implementation, distribution, review, revision, and archival of all critical documents.(17)	Yes	Yes	Yes
**4.2.3**	The Quality Management Plan **shall** include, or summarize and reference, policies and SOPs for the establishment and maintenance of written agreements.(17)	Yes	Yes	Yes
**4.2.3.1**	Agreements **shall** be established with external parties (who are accredited, as appropriate) providing critical services that could affect the quality and safety of care for patients in the Clinical Program.(17)	Yes	Yes	Yes
**4.2.3.2**	Agreements **shall** be dated and reviewed on a regular basis.(14)	Yes	Yes	Yes
**4.2.4**	The Quality Management Plan **shall** include, or summarize and reference, policies and SOPs for occurrences including near misses, errors, accidents, deviations, adverse events, adverse reactions, and complaints. This maybe the same as existing policy at the centre.(17)	Yes	Yes	Yes
**4.2.5**	The Quality Management Plan **shall** include, or summarize and reference, policies and SOPs for actions to take in the event the Clinical Program’s operations are interrupted.(17)	Yes	Yes	Yes
**4.2.6**	The Quality Management Plan **shall** include, or summarize and reference, policies and SOPs for qualification of critical manufacturers, vendors, equipment, supplies, reagents, facilities, and services.(17)	Yes	Yes	Yes

#	Recommendation	Transplant and Acute Leukemia Service Site	Acute Leukemia Service Site	Shared-Care Partner Centre
**4.2.6.1**	Qualification plans, results, and reports **shall** be reviewed and approved by the Quality Manager and Clinical Program Medical Director or designate.(17)	Yes	Yes	Yes
**4.2.7**	The Quality Management Plan **shall** include, or summarize and reference, policies and SOPs for the evaluation of risk in changes to a process to confirm that the changes do not create an adverse impact or inherent risk elsewhere in the operation.(17)	Yes	Yes	Yes
**4.2.8**	The Quality Management Plan **shall** include, or summarize and reference, an organizational chart of key positions and functions within the Clinical Program (governance structure).(17)	Yes	Yes	Yes
**4.2.8.1**	The Quality Management Plan **shall** include a description of how these key positions interact to implement the quality management activities.(17)	Yes	Yes	Yes
**4.2.8.2**	The Quality Management Plan **shall** include, or summarize and reference, policies and SOPs addressing personnel requirements for each key position in the Clinical Program. Personnel requirements shall include at a minimum:	Yes	Yes	Yes
**4.2.8.2.1**	A current job description for all staff.(17)	Yes	Yes	Yes
**4.2.8.2.2**	A system to document the following for all staff:<br>• Initial qualifications<br>• New employee orientation<br>• Initial training, competency, and retraining when appropriate for all procedures performed<br>• Continued competency for each critical function performed, assessed annually at a minimum Continuing education.(17)	Yes	Yes	Yes
**4.2.9**	The Quality Management Plan **shall** include key performance indicators and outcome analysis.(14)	Yes	Yes	Yes
**4.2.9.1**	The Clinical Program **should** work with Ontario Health (Cancer Care Ontario) to meet Provincial Acute Leukemia Program benchmarks, including:	Yes	Yes	Yes
**4.2.9.1.1**	Consult Wait Times	Yes	Yes	No
**4.2.9.1.2**	Length of stay	Yes	Yes	Yes
**4.2.9.1.3**	Mortality	Yes	Yes	Yes
**4.2.9.1.4**	Survival Outcomes	Yes	Yes	Yes
**4.2.9.1.5**	Treatment Utilization	Yes	Yes	Yes

#	Recommendation	Transplant and Acute Leukemia Service Site	Acute Leukemia Service Site	Shared-Care Partner Centre
**4.2.9.1.6**	Treatment Wait Times and	Yes	Yes	No
**4.2.9.1.7**	Other indicators, as established.(14)	Yes	Yes	Yes
**4.2.9.2**	In addition to the Ontario Health (Cancer Care Ontario) recommended metrics, review of outcome analysis **shall** include at a minimum:	Yes	Yes	Yes
**4.2.9.2.1**	Central venous catheter infection and/or thrombosis	Yes	Yes	Yes
**4.2.9.2.2**	Complete remission	Yes	Yes	Yes
**4.2.9.2.3**	Hospital acquired infections	Yes	Yes	Yes
**4.2.9.2.4**	ICU admissions.(14,17)	Yes	Yes	Yes
**4.2.10**	The Quality Management Plan **shall** include, or summarize and reference, policies and SOPs for, and a schedule of, audits of the Clinical Program's activities to verify compliance with elements of the Quality Management Program and policies and SOPs, applicable laws or regulations, and these Specifications.(17)	Yes	Yes	Yes
**4.2.10.1**	The results of audits **shall** be used to recognize problems, detect trends, identify improvement opportunities, and implement corrective and preventive actions, when necessary, and follow-up on the effectiveness of these actions in a timely manner.(17)	Yes	Yes	Yes
**4.2.10.2**	Audits **shall** be conducted by an individual with sufficient expertise to identify problems, but who is not solely responsible for the process being audited.(17)	Yes	Yes	Yes
**POLICIES AND PROCEDURES**				
**5.1**	The Centre **shall** have SOPs that are detailed, as per hospital's policy, to allow qualified staff to follow and complete the procedures successfully.(14)	Yes	Yes	Yes
**5.2**	The Clinical Program **shall** have SOPs defining local protocols for patient eligibility and selection for care (including performance status, prognostic factors, comorbidities) and consent.(11,17)	Yes	Yes	Yes
**5.3**	There **shall** be written SOPs or guidelines, including, but not limited to:	Yes	Yes	Yes
**5.3.1**	All clinical procedures	Yes	Yes	Yes
**5.3.2**	Administration of systemic therapy	Yes	Yes	Yes
**5.3.3**	Central venous access device care	Yes	Yes	Yes
**5.3.4**	Management of complications with systemic therapy:	Yes	Yes	Yes
**5.3.4.1**	Nausea, vomiting, pain, and other discomforts	Yes	Yes	Yes

#	Recommendation	Transplant and Acute Leukemia Service Site	Acute Leukemia Service Site	Shared-Care Partner Centre
**5.3.4.2**	Monitoring of blood counts and transfusion of blood products	Yes	Yes	Yes
**5.3.4.3**	Monitoring and management of infections and use of antimicrobials	Yes	Yes	Yes
**5.3.4.4**	Monitoring of organ dysfunction or failure	Yes	Yes	Yes
**5.3.5**	Prophylaxis, management and care of immunocompromised patients.(11,14,17,24)	Yes	Yes	Yes
**5.4**	The Centre **shall** have policies addressing safe administration of patient-specific radiation therapy.(25)	Yes	Yes	Yes
**5.5**	The Clinical Program **shall** have policies or SOPs in place for planned discharges and provision of follow-up care post-systemic therapy care, including transfer of patient, if required.(17)	Yes	Yes	Yes
**5.6**	The Clinical Program **shall** have a SOP for inter-institutional patient transfer that specifies clinical criteria for eligibility to transfer the patient and information transferred with the patient.(14)	Yes	Yes	Yes
**5.7**	The Clinical Program **shall** have an SOP for electronic decision-making tools used by the Clinical Program documenting the tools development, validation and auditing.(14)	Yes	Yes	Yes
**5.8**	Staff training and, if appropriate, competency **shall** be documented before performing a new or revised SOP or guideline.(17)	Yes	Yes	Yes
**5.9**	Planned deviations from SOPs **shall** be pre-approved by the Clinical Program Medical Director, or designate, and reviewed by the Quality Manager.(17)	Yes	Yes	Yes
**5.10**	The Centre **should** have a SOP for the recognition of systemic therapy-related complications and emergencies requiring rapid notification of the Clinical Program.(17)	Yes	Yes	Yes
**5.11**	The Centre **should** have an institutional SOP for direct admission of patients to the hematology ward or other facilities equipped to rapidly assess and manage potentially life-threatening complications of systemic therapy (such as neutropenic sepsis or bleeding), where appropriate.(11)	Yes	Yes	Yes
**5.12**	The Clinical Program **should** have an established framework or policies for the transfer of patients to the ICU, as appropriate. The framework/policies **should** include written guidelines for communication, patient monitoring, and prompt triage or transfer of patients to an ICU when appropriate.(17,26)	Yes	Yes	Yes

#	Recommendation	Transplant and Acute Leukemia Service Site	Acute Leukemia Service Site	Shared-Care Partner Centre
**5.13**	The Centre **should** have written policies for communication with the person's primary care physician and other teams involved in treatment.(11)	Yes	Yes	Yes
**PATIENT CARE**				
**6.1**	The Clinical Program **shall** obtain patient informed consent, as per Accreditation Canada, for systemic therapy, which is documented in the patient's medical record by a licensed health care professional familiar with the proposed systemic therapy.(7,14)	Yes	Yes	Yes
**6.1.1**	The Clinical Program **shall** provide information regarding the risks, benefits, and alternatives of the proposed systemic therapy.(17)	Yes	Yes	Yes
**6.1.2**	The Centre **shall** provide the patient with information regarding the impact of treatment on fertility and information, including contact information, about fertility preservation.(14)	Yes	Yes	Yes
**6.2**	The Centre **shall** provide the patient with access to active palliative care, supportive end of life care(27) and medical assistance in dying (MAID).(28) In accordance with patient and family's wishes, this care could be provided at centres at and beyond the acute leukemia service provider sites and can be offered closer to home.(2)	Yes	Yes	Yes
**6.3**	If radiation is used, the centre **shall** document a final report with details of the radiation therapy administered in the patient's medical record that is accessible to the acute leukemia team.(17)	Yes	Yes	Yes
**6.4**	The Clinical Program **shall** provide services for adolescents and young adult patients and a process describing the transition and acceptance of adolescents and young adult patients to adult care, as appropriate.(12,17)	Yes	Yes	Yes
**6.5**	The Centre **should** provide formal Multidisciplinary Case Conferences (MCC), where acute leukemia cases may be presented and discussed, attended by individuals detailed in the Cancer Care Ontario's MCC Standards.(11,29)	Yes	Yes	Yes
**6.6**	The Clinical Program **should** provide acute leukemia patients with access to a designated contact person, as part of a multidisciplinary team, throughout the duration of their care.(30)	Yes	Yes	Yes
**6.7**	The Centre **should** provide care in alignment with Cancer Care Ontario's Person-Centred Care Guidelines(31), in efforts to meet the Person-Centred goals and objectives detailed in the most recent version of the Ontario Cancer Plan.(32)	Yes	Yes	Yes

#	Recommendation	Transplant and Acute Leukemia Service Site	Acute Leukemia Service Site	Shared-Care Partner Centre
**CLINICAL RESEARCH**				
**7.1**	The Centre **shall** conduct and/or provide access to clinical trials and consider available clinical trials when assessing patient treatment options.(2,23)	Yes	Yes	No
**7.2**	The Centre **shall** report available clinical trials for patients with acute leukemia to Ontario Health (Cancer Care Ontario) (by emailing OH-CCO_SSOInfo@ontariohealth.ca) or a central repository to inform other centres of their availability.(14)	Yes	Yes	No
**DATA MANAGEMENT**				
**8.1**	The Centre **shall** be compliant with laws and regulations regarding storage and use of personal health information as detailed by the Information and Privacy Commissioner of Ontario.(14,15)	Yes	Yes	Yes
**8.2**	The Centre **shall** be compliant with Cancer Care Ontario Data Book.(2)	Yes	Yes	Yes
**8.3**	The Clinical Program **shall** collect all the data necessary to complete data submission requirements of Ontario Health (Cancer Care Ontario), as detailed in the Funding Agreement with Ontario Health (Cancer Care Ontario).(14)	Yes	Yes	Yes
**8.4**	The Clinical Program **should** have an IT system that allows:	Yes	Yes	Yes
**8.4.1**	Specimen booking and registration at source	Yes	Yes	Yes
**8.4.2**	Input and update of clinical information	Yes	Yes	Yes
**8.4.3**	Integrated/synoptic reporting	Yes	Yes	Yes
**8.4.4**	Secure internal and external two-way communication between health care professional.(11)	Yes	Yes	Yes
**8.5**	Defined data management staff **should** participate in continuing education annually.(17)	Yes	Yes	Yes
**LABORATORY SERVICES**				
**9.1**	The Centre **shall** ensure patients have access to all required pathology and molecular diagnostic tests as listed in the most recent version of the Consensus Pathology Recommendation for Complex Malignant Hematology Report [25].	Yes	Yes	Yes
**9.1.1**	Testing sites **shall** meet all relevant Institute for Quality Management in Healthcare requirements and maintain Institute for Quality Management certification.(14)	Yes	Yes	Yes
**9.1.2**	All testing performed for clinical management **should** be licensed and performed by accredited labs.(14)	Yes	Yes	Yes

#	Recommendation	Transplant and Acute Leukemia Service Site	Acute Leukemia Service Site	Shared-Care Partner Centre
**9.1.3**	Testing sites performing cytogenetics and molecular diagnostics **shall** meet provincial turnaround time targets.(33)	Yes	Yes	Yes
**9.2**	The Centre **shall** classify and report acute leukemia and subtypes based on the current World Health Organization classification system and Ontario Health (Cancer Care Ontario) Synoptic Reporting, when in place.(11,33,34)	Yes	Yes	Yes
